# 1-(4-Chloro-2-fluoro-5-nitro­phen­yl)-4-difluoro­methyl-3-methyl-1*H*-1,2,4-triazol-5(4*H*)-one

**DOI:** 10.1107/S1600536812013013

**Published:** 2012-04-13

**Authors:** Li Yang, Jun Liu

**Affiliations:** a Henan Medical College for Staff and Workers, Zhengzhou 451191, People’s Republic of China

## Abstract

In the title compound, C_10_H_6_ClF_3_N_4_O_3_, the dihedral angle between the benzene ring and the triazolone ring is 59.9 (1)°, while the nitro substituent subtends an angle of 39.5 (1)° to the benzene ring plane. In the crystal, pairs of mol­ecules form inversion dimers *via* C—H⋯O hydrogen bonds.

## Related literature

For background to applications of this class of compound, see: Ager & Polsz (1996 ▶). For the synthesis and the use of the title compound in the production of herbicides, see: Goudar (1998 ▶). For bond-length data, see: Allen *et al.* (1987 ▶).
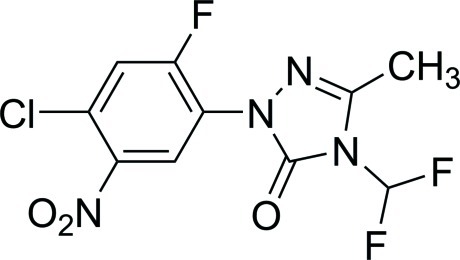


## Experimental

### 

#### Crystal data

C_10_H_6_ClF_3_N_4_O_3_
*M* *_r_* = 322.64
Monoclinic, 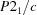

*a* = 12.556 (3) Å
*b* = 14.800 (3) Å
*c* = 6.8760 (14) Å
β = 103.32 (3)°
*V* = 1243.4 (4) Å^3^
*Z* = 4
Mo *K*α radiation
μ = 0.36 mm^−1^
*T* = 293 K
0.30 × 0.20 × 0.10 mm

#### Data collection

Enraf–Nonius CAD-4 diffractometer
Absorption correction: ψ scan (North *et al.*, 1968 ▶) *T*_min_ = 0.899, *T*_max_ = 0.965
4877 measured reflections
2293 independent reflections
1589 reflections with *I* > 2σ(*I*)
*R*_int_ = 0.073
3 standard reflections every 200 reflections intensity decay: 1%

#### Refinement

*R*[*F* ^2^ > 2σ(*F* ^2^)] = 0.068
*wR*(*F* ^2^) = 0.185
*S* = 1.01
2293 reflections
190 parameters
H-atom parameters constrained
Δρ_max_ = 0.78 e Å^−3^
Δρ_min_ = −0.34 e Å^−3^

### 

Data collection: *CAD-4 Software* (Enraf–Nonius, 1985 ▶); cell refinement: *CAD-4 Software*; data reduction: *XCAD4* (Harms & Wocadlo, 1995 ▶); program(s) used to solve structure: *SHELXS97* (Sheldrick, 2008 ▶); program(s) used to refine structure: *SHELXS97* (Sheldrick, 2008 ▶); molecular graphics: *SHELXTL* (Sheldrick, 2008 ▶); software used to prepare material for publication: *SHELXTL*.

## Supplementary Material

Crystal structure: contains datablock(s) I, global. DOI: 10.1107/S1600536812013013/sj5217sup1.cif

Structure factors: contains datablock(s) I. DOI: 10.1107/S1600536812013013/sj5217Isup2.hkl

Supplementary material file. DOI: 10.1107/S1600536812013013/sj5217Isup3.cml

Additional supplementary materials:  crystallographic information; 3D view; checkCIF report

## Figures and Tables

**Table 1:** Hydrogen-bond geometry (Å, °)

*D*—H⋯*A*	*D*—H	H⋯*A*	*D*⋯*A*	*D*—H⋯*A*
C10—H10*A*⋯O1^i^	0.98	2.32	3.190 (6)	148

Symmetry code: (i) 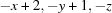.

**Fig. 1.**
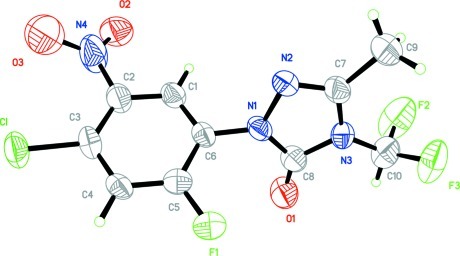
The molecular structure of (I), with the atom-numbering scheme. Displacement ellipsoids are drawn at the 50% probability level.

**Fig. 2.**
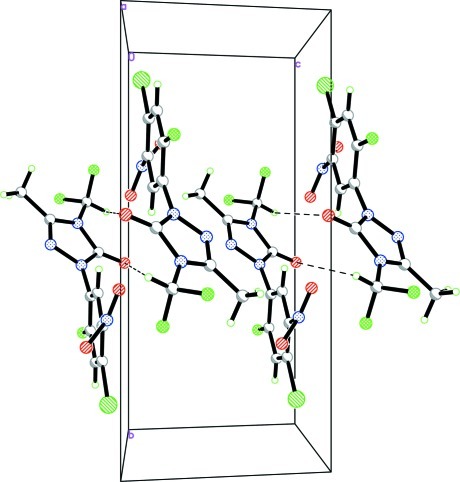
A packing diagram for (I) with hydrogen bonds drawn as dashed lines.

## supplementary crystallographic information

### Comment

The title compound is an important intermediate used to synthesize the herbicide Carfentrazone-ethyl. It can also be used to synthesize other herbicides (Goudar, 1998), which are of wide interest for application to the control of broadleaf weeds and sedges (Ager & Polsz, 1996). We report here the crystal structure of the title compound, (I), which is of interest to us in this field.

The molecular structure of (I) is shown in Fig. 1. Bond distances in the molecule are normal (Allen *et al.*, 1987). The dihedral angle between the C1—C6 and N1/N3/C8/N2/C7 rings is 59.9 (1)° and the nitro substituent subtends an angle of 39.5 (1)° to the benzene ring plane.. In the crystal structure, molecules form inversion dimers via intermolecular C10—H10A···O1 hydrogen bonds (Table 1, Fig 2) .

### Experimental

The title compound, (I) was prepared by a method reported in literature (Goudar, 1998). Crystals were obtained by dissolving (I) (0.2 g) in acetone (50 ml) and evaporating the solvent slowly at room temperature over 10 d.

### Refinement

All H atoms were positioned geometrically and constrained to ride on their parent atoms, with C—H = 0.93 Å for aromatic H and 0.96 Å for alkyl H, respectively. The *U*_iso_(H) = *xU*_eq_(C), where *x* = 1.2 for aromatic H, and *x* = 1.5 for alkyl H.

### Crystal data

**Table:**

C_10_H_6_ClF_3_N_4_O_3_	*F*(000) = 648
*M**_r_* = 322.64	*D*_x_ = 1.724 Mg m^−^^3^
Monoclinic, *P*2_1_/*c*	Mo *K*α radiation, λ = 0.71073 Å
Hall symbol: -P 2ybc	Cell parameters from 25 reflections
*a* = 12.556 (3) Å	θ = 9–13°
*b* = 14.800 (3) Å	µ = 0.36 mm^−^^1^
*c* = 6.8760 (14) Å	*T* = 293 K
β = 103.32 (3)°	Block, colourless
*V* = 1243.4 (4) Å^3^	0.30 × 0.20 × 0.10 mm
*Z* = 4	

### Data collection

**Table:**

Enraf–Nonius CAD-4 diffractometer	1589 reflections with *I* > 2σ(*I*)
Radiation source: fine-focus sealed tube	*R*_int_ = 0.073
Graphite monochromator	θ_max_ = 25.4°, θ_min_ = 1.7°
ω/2θ scans	*h* = −15→14
Absorption correction: ψ scan (North *et al.*, 1968)	*k* = −17→17
*T*_min_ = 0.899, *T*_max_ = 0.965	*l* = 0→8
4877 measured reflections	3 standard reflections every 200 reflections
2293 independent reflections	intensity decay: 1%

### Refinement

**Table:**

Refinement on *F*^2^	Primary atom site location: structure-invariant direct methods
Least-squares matrix: full	Secondary atom site location: difference Fourier map
*R*[*F*^2^ > 2σ(*F*^2^)] = 0.068	Hydrogen site location: inferred from neighbouring sites
*wR*(*F*^2^) = 0.185	H-atom parameters constrained
*S* = 1.01	*w* = 1/[σ^2^(*F*_o_^2^) + (0.1*P*)^2^ + 0.330*P*] where *P* = (*F*_o_^2^ + 2*F*_c_^2^)/3
2293 reflections	(Δ/σ)_max_ < 0.001
190 parameters	Δρ_max_ = 0.78 e Å^−^^3^
0 restraints	Δρ_min_ = −0.34 e Å^−^^3^

### Special details

**Table:**

Geometry. All e.s.d.'s (except the e.s.d. in the dihedral angle between two l.s. planes) are estimated using the full covariance matrix. The cell e.s.d.'s are taken into account individually in the estimation of e.s.d.'s in distances, angles and torsion angles; correlations between e.s.d.'s in cell parameters are only used when they are defined by crystal symmetry. An approximate (isotropic) treatment of cell e.s.d.'s is used for estimating e.s.d.'s involving l.s. planes.
Refinement. Refinement of *F*^2^ against ALL reflections. The weighted *R*-factor *wR* and goodness of fit *S* are based on *F*^2^, conventional *R*-factors *R* are based on *F*, with *F* set to zero for negative *F*^2^. The threshold expression of *F*^2^ > σ(*F*^2^) is used only for calculating *R*-factors(gt) *etc*. and is not relevant to the choice of reflections for refinement. *R*-factors based on *F*^2^ are statistically about twice as large as those based on *F*, and *R*- factors based on ALL data will be even larger.

### Fractional atomic coordinates and isotropic or equivalent isotropic displacement parameters (Å^2^)

**Table:**

	*x*	*y*	*z*	*U*_iso_*/*U*_eq_	
Cl	0.47555 (10)	0.11625 (7)	0.08507 (17)	0.0670 (4)	
F1	0.84029 (19)	0.26681 (16)	0.2492 (4)	0.0710 (7)	
C1	0.5785 (3)	0.3747 (2)	0.1483 (5)	0.0470 (9)	
H1A	0.5481	0.4322	0.1386	0.056*	
N1	0.7581 (2)	0.43972 (19)	0.2491 (4)	0.0444 (7)	
O1	0.8646 (3)	0.4432 (2)	0.0175 (5)	0.0709 (9)	
O2	0.3580 (3)	0.3887 (3)	0.0249 (5)	0.0798 (10)	
C2	0.5118 (3)	0.2996 (3)	0.1173 (7)	0.0580 (10)	
F2	0.9214 (3)	0.6832 (2)	0.1945 (7)	0.1216 (14)	
N2	0.7474 (2)	0.4912 (2)	0.4138 (5)	0.0481 (8)	
N3	0.8789 (2)	0.5451 (2)	0.2821 (5)	0.0469 (8)	
C3	0.5549 (3)	0.2129 (2)	0.1239 (6)	0.0521 (9)	
F3	1.0420 (2)	0.6114 (2)	0.4004 (5)	0.0862 (9)	
C4	0.6663 (3)	0.2027 (2)	0.1651 (5)	0.0414 (8)	
H4A	0.6970	0.1454	0.1694	0.050*	
N4	0.3939 (4)	0.3183 (3)	0.0870 (10)	0.113 (2)	
C5	0.7323 (3)	0.2774 (2)	0.2001 (5)	0.0408 (8)	
C6	0.6898 (3)	0.3641 (2)	0.1934 (5)	0.0379 (8)	
C7	0.8207 (3)	0.5538 (2)	0.4266 (6)	0.0476 (9)	
C8	0.8380 (3)	0.4709 (2)	0.1627 (5)	0.0471 (9)	
C9	0.8423 (4)	0.6223 (3)	0.5868 (8)	0.0770 (14)	
H9A	0.7916	0.6145	0.6708	0.116*	
H9B	0.8337	0.6816	0.5287	0.116*	
H9C	0.9157	0.6153	0.6653	0.116*	
C10	0.9601 (3)	0.6014 (3)	0.2389 (7)	0.0626 (12)	
H10A	0.9878	0.5764	0.1281	0.075*	
O3	0.3405 (3)	0.2597 (4)	0.1869 (10)	0.143 (2)	

### Atomic displacement parameters (Å^2^)

**Table:**

	*U*^11^	*U*^22^	*U*^33^	*U*^12^	*U*^13^	*U*^23^
Cl	0.0777 (8)	0.0436 (6)	0.0810 (8)	−0.0207 (5)	0.0213 (6)	−0.0081 (5)
F1	0.0482 (13)	0.0551 (15)	0.109 (2)	0.0087 (11)	0.0167 (13)	−0.0066 (14)
C1	0.054 (2)	0.0352 (19)	0.053 (2)	0.0013 (16)	0.0148 (18)	0.0006 (15)
N1	0.0564 (18)	0.0368 (16)	0.0461 (16)	−0.0068 (14)	0.0245 (14)	−0.0097 (13)
O1	0.083 (2)	0.071 (2)	0.0702 (18)	−0.0221 (17)	0.0428 (17)	−0.0113 (16)
O2	0.062 (2)	0.077 (2)	0.097 (2)	0.0182 (17)	0.0115 (18)	0.0012 (19)
C2	0.045 (2)	0.042 (2)	0.081 (3)	−0.0005 (17)	0.003 (2)	−0.0040 (19)
F2	0.078 (2)	0.0610 (19)	0.216 (4)	−0.0003 (15)	0.015 (2)	0.064 (2)
N2	0.0489 (18)	0.0391 (17)	0.0596 (19)	−0.0017 (14)	0.0191 (15)	−0.0098 (14)
N3	0.0440 (17)	0.0391 (17)	0.0594 (18)	−0.0089 (13)	0.0157 (15)	−0.0011 (14)
C3	0.060 (2)	0.036 (2)	0.057 (2)	−0.0128 (18)	0.0075 (18)	−0.0031 (16)
F3	0.0552 (16)	0.085 (2)	0.107 (2)	−0.0209 (14)	−0.0060 (15)	0.0192 (16)
C4	0.064 (2)	0.0314 (17)	0.0366 (17)	0.0015 (16)	0.0283 (16)	−0.0008 (14)
N4	0.047 (2)	0.061 (3)	0.221 (6)	−0.007 (2)	0.011 (3)	−0.023 (4)
C5	0.046 (2)	0.047 (2)	0.0308 (16)	0.0043 (16)	0.0127 (14)	0.0011 (14)
C6	0.056 (2)	0.0337 (18)	0.0290 (16)	−0.0064 (15)	0.0188 (15)	−0.0035 (12)
C7	0.046 (2)	0.0327 (18)	0.060 (2)	0.0052 (16)	0.0041 (18)	−0.0031 (16)
C8	0.059 (2)	0.042 (2)	0.0453 (19)	−0.0057 (17)	0.0238 (18)	0.0005 (16)
C9	0.066 (3)	0.056 (3)	0.110 (4)	−0.002 (2)	0.021 (3)	−0.031 (3)
C10	0.059 (3)	0.040 (2)	0.089 (3)	−0.0079 (19)	0.017 (2)	0.023 (2)
O3	0.066 (2)	0.120 (4)	0.248 (7)	−0.023 (3)	0.044 (3)	−0.065 (4)

### Geometric parameters (Å, º)

**Table:**

Cl—C3	1.729 (4)	N3—C7	1.368 (5)
F1—C5	1.329 (4)	N3—C8	1.397 (5)
C1—C6	1.368 (5)	N3—C10	1.401 (5)
C1—C2	1.379 (5)	C3—C4	1.370 (5)
C1—H1A	0.9300	F3—C10	1.336 (5)
N1—C8	1.359 (5)	C4—C5	1.369 (5)
N1—N2	1.397 (4)	C4—H4A	0.9300
N1—C6	1.408 (4)	N4—O3	1.373 (8)
O1—C8	1.196 (4)	C5—C6	1.387 (5)
O2—N4	1.176 (6)	C7—C9	1.475 (6)
C2—C3	1.389 (5)	C9—H9A	0.9600
C2—N4	1.473 (6)	C9—H9B	0.9600
F2—C10	1.315 (5)	C9—H9C	0.9600
N2—C7	1.294 (5)	C10—H10A	0.9800
			
C6—C1—C2	119.7 (3)	F1—C5—C6	118.7 (3)
C6—C1—H1A	120.1	C4—C5—C6	121.9 (3)
C2—C1—H1A	120.1	C1—C6—C5	118.6 (3)
C8—N1—N2	112.8 (3)	C1—C6—N1	119.8 (3)
C8—N1—C6	128.0 (3)	C5—C6—N1	121.3 (3)
N2—N1—C6	119.2 (3)	N2—C7—N3	111.9 (3)
C1—C2—C3	121.4 (4)	N2—C7—C9	123.3 (4)
C1—C2—N4	115.2 (4)	N3—C7—C9	124.7 (4)
C3—C2—N4	123.3 (4)	O1—C8—N1	128.7 (4)
C7—N2—N1	104.3 (3)	O1—C8—N3	128.7 (3)
C7—N3—C8	108.4 (3)	N1—C8—N3	102.6 (3)
C7—N3—C10	129.4 (3)	C7—C9—H9A	109.5
C8—N3—C10	122.0 (3)	C7—C9—H9B	109.5
C4—C3—C2	118.7 (3)	H9A—C9—H9B	109.5
C4—C3—Cl	117.7 (3)	C7—C9—H9C	109.5
C2—C3—Cl	123.5 (3)	H9A—C9—H9C	109.5
C5—C4—C3	119.7 (3)	H9B—C9—H9C	109.5
C5—C4—H4A	120.2	F2—C10—F3	105.3 (4)
C3—C4—H4A	120.2	F2—C10—N3	110.4 (4)
O2—N4—O3	123.4 (5)	F3—C10—N3	110.4 (3)
O2—N4—C2	120.4 (5)	F2—C10—H10A	110.2
O3—N4—C2	113.7 (5)	F3—C10—H10A	110.2
F1—C5—C4	119.4 (3)	N3—C10—H10A	110.2
			
C6—C1—C2—C3	−2.3 (6)	C8—N1—C6—C1	−123.1 (4)
C6—C1—C2—N4	174.9 (4)	N2—N1—C6—C1	57.8 (4)
C8—N1—N2—C7	0.3 (4)	C8—N1—C6—C5	62.6 (5)
C6—N1—N2—C7	179.5 (3)	N2—N1—C6—C5	−116.5 (3)
C1—C2—C3—C4	1.0 (6)	N1—N2—C7—N3	−0.9 (4)
N4—C2—C3—C4	−176.0 (5)	N1—N2—C7—C9	−177.2 (4)
C1—C2—C3—Cl	−179.7 (3)	C8—N3—C7—N2	1.2 (4)
N4—C2—C3—Cl	3.4 (7)	C10—N3—C7—N2	176.2 (4)
C2—C3—C4—C5	0.5 (6)	C8—N3—C7—C9	177.5 (4)
Cl—C3—C4—C5	−178.9 (2)	C10—N3—C7—C9	−7.5 (6)
C1—C2—N4—O2	25.3 (8)	N2—N1—C8—O1	−177.8 (4)
C3—C2—N4—O2	−157.6 (5)	C6—N1—C8—O1	3.1 (7)
C1—C2—N4—O3	−137.4 (5)	N2—N1—C8—N3	0.4 (4)
C3—C2—N4—O3	39.7 (8)	C6—N1—C8—N3	−178.7 (3)
C3—C4—C5—F1	176.9 (3)	C7—N3—C8—O1	177.3 (4)
C3—C4—C5—C6	−0.6 (5)	C10—N3—C8—O1	1.9 (6)
C2—C1—C6—C5	2.1 (5)	C7—N3—C8—N1	−0.9 (4)
C2—C1—C6—N1	−172.3 (3)	C10—N3—C8—N1	−176.3 (3)
F1—C5—C6—C1	−178.2 (3)	C7—N3—C10—F2	−59.3 (6)
C4—C5—C6—C1	−0.7 (5)	C8—N3—C10—F2	115.1 (5)
F1—C5—C6—N1	−3.9 (4)	C7—N3—C10—F3	56.7 (5)
C4—C5—C6—N1	173.6 (3)	C8—N3—C10—F3	−128.9 (4)

### Hydrogen-bond geometry (Å, º)

**Table:**

*D*—H···*A*	*D*—H	H···*A*	*D*···*A*	*D*—H···*A*
C10—H10*A*···O1^i^	0.98	2.32	3.190 (6)	148

Symmetry code: (i) −*x*+2, −*y*+1, −*z*.
